# Clinical predictors of pathological gastroesophageal reflux: a pH-impedance study of 2688 adults with laryngopharyngeal reflux symptoms

**DOI:** 10.1093/dote/doag089

**Published:** 2026-08-12

**Authors:** Luis Fernando Pineda Ovalle, Valentina Salcedo, Alejandradel Pilar Rodríguez Orjuela, Cristian Andrés Mora León, Andrea Gómez Rodríguez, Laura Moya Valenzuela, David Páramo Hernández

**Affiliations:** GutMédica Center, Institute of Digestive Health, Bogotá, Colombia; Faculty of Medicine, Universidad Nacional de Colombia (UNAL), Bogotá, Colombia; GutMédica Center, Institute of Digestive Health, Bogotá, Colombia; School of Medicine and Health Sciences, Faculty of Medicine, Universidad del Rosario, Bogotá, Colombia; GutMédica Center, Institute of Digestive Health, Bogotá, Colombia; School of Medicine and Health Sciences, Faculty of Medicine, Universidad del Rosario, Bogotá, Colombia; Faculty of Medicine, Universidad Nacional de Colombia (UNAL), Bogotá, Colombia; GutMédica Center, Institute of Digestive Health, Bogotá, Colombia; School of Medicine and Health Sciences, Faculty of Medicine, Universidad del Rosario, Bogotá, Colombia; GutMédica Center, Institute of Digestive Health, Bogotá, Colombia; School of Medicine and Health Sciences, Faculty of Medicine, Universidad del Rosario, Bogotá, Colombia; GutMédica Center, Institute of Digestive Health, Bogotá, Colombia; School of Medicine and Health Sciences, Faculty of Medicine, Universidad del Rosario, Bogotá, Colombia; GutMédica Center, Institute of Digestive Health, Bogotá, Colombia; Faculty of Medicine, Universidad Nacional de Colombia (UNAL), Bogotá, Colombia

**Keywords:** esophageal pH monitoring, gastroesophageal reflux, laryngopharyngeal reflux

## Abstract

Gastroesophageal reflux disease (GERD) is implicated as a potential cause of chronic laryngopharyngeal symptoms, including laryngitis, cough, dysphonia, and throat clearing, often grouped under laryngopharyngeal reflux (LPR). This study aimed to assess the predictive value of clinical and demographic factors for GERD in patients with LPR symptoms using multichannel intraluminal impedance–pH monitoring. Analytical cross-sectional study of 2688 adults with upper airway symptoms and suspected LPR undergoing pH-impedance monitoring between January 2019 and December 2022. Pathological GERD was defined as acid exposure time (AET) >6%, or AET 4%–6% with abnormal reflux parameters (total reflux events >80 and/or positive symptom association). Proximal reflux episodes were recorded but not used diagnostically. Multivariable logistic regression analyses identified independent predictors. Among 2688 patients (73.6% female; mean age 54.5 years), only 8.4% had pathological GERD. Independent predictors included higher body mass index, male sex, older age, heartburn, and hiatal hernia. The final model demonstrated moderate discrimination area under the curve (AUC) 0.676. Only a small proportion of patients with suspected LPR have objective GERD. Pathological reflux is better predicted by traditional GERD-related factors than by isolated laryngopharyngeal symptoms, supporting a more cautious interpretation of LPR and consideration of alternative diagnoses.

## INTRODUCTION

Gastroesophageal reflux disease (GERD) is a highly prevalent condition worldwide. According to an expert consensus, GERD is subdivided into two major groups: esophageal syndromes and extraesophageal syndromes.^1^ The first group includes patients with typical symptoms such as heartburn, regurgitation, and chest pain and/or the presence of specific esophageal lesions such as erosive esophagitis, peptic strictures, Barrett’s esophagus, and esophageal adenocarcinoma. The second group includes the extraesophageal syndromes, which compromises patients with atypical symptoms such as laryngitis, cough, dysphonia, postnasal drip, asthma, and dental erosions.^1^

Laryngopharyngeal reflux (LPR) is physiopathologically defined as the retrograde flow of gastric contents into the laryngopharynx, where it encounters the tissues of the upper aerodigestive tract, generating a secondary inflammatory response.^2^^,^^3^ Most symptoms observed in LPR such as cough, odynophagia, throat clearing, and globus sensation can also be seen in other clinical conditions, including allergic, infectious, and autoimmune diseases, among others.^3–7^ Consequently, implementing diagnostic and therapeutic approach in these patients is crucial to avoid inappropriate and expensive management. The cost of treating LPR is high, approximately 50 billion USD per year, representing expenses 5.6 times higher than those associated with typical GERD.^5^^,^^6^ This is largely due to an imbalance between the high reported prevalence of the disease and the difficulty in establishing precise diagnostic criteria, scoring systems, and methods to confirm it. Despite these limitations, primary care physicians, otolaryngologists, and gastroenterologists frequently attribute multiple laryngopharyngeal symptoms to GERD, even in the absence of evidence demonstrating the presence of the disease.^4^ LPR continues to be a very common diagnosis in the general population: epidemiological studies using symptoms as diagnostic criteria have reported a prevalence of 20%–60% in the United States, and it accounts for approximately 10% of otolaryngology consultations.^2^^,^^3^ For all these reasons, the clinical management of patients with suspected LPR represents an important challenge for treating physicians, as there is no diagnostic gold standard, and symptoms are irregular, with low sensitivity and specificity.^8^

The objective of this study is to determine the predictive capacity of clinical variables (symptoms and demographic data) for the diagnosis of pathologic GERD in a large cohort of patients with LPR symptoms, using esophageal multichannel intraluminal impedance–pH monitoring as the comparative objective reference for GERD. In addition, the study aims to establish the prevalence of objective GERD in this patient group. Based on the results of this study, more reasoned decisions can be made regarding the use of confirmatory diagnostic testing for GERD or the initiation of empirical therapy in patients with suspected LPR.

## METHODS

This analytical cross-sectional observational study was conducted using retrospectively collected data from patients evaluated at a referral center for gastrointestinal physiology and motility testing. Although data were collected between January 2019 and December 2022, each patient contributed a single observation, with exposures and outcomes assessed at the time of pH-impedance monitoring. Clinical, demographic, and pH-impedance data were assessed from 2688 patients referred to our service for reflux monitoring due to suspected LPR between January 2019 and December 2022. All reflux monitoring studies included in this analysis were performed using standard 24-hour ambulatory catheter-based pH-impedance monitoring.

Inclusion criteria were adults ≥18 years of age, referral for upper airway symptoms requiring pH-impedance monitoring, and no use of proton pump inhibitors (PPIs) or histamine H2-receptor antagonists during the 10 days prior to testing. Exclusion criteria included failure to meet inclusion criteria, presence of upper airway lesions, head and neck tumors, prior gastric or esophageal surgery, known esophageal motility disorders (including achalasia, absent contractility, and spastic disorders), or incomplete registry data for variables relevant to the study. The final sample size was obtained after applying these criteria to more than 4000 initial records.

The dependent variable was pathological reflux, treated as a dichotomous outcome and it was defined as acid exposure time (AET) >6%, or alternatively, AET between 4% and 6% in combination with either a total number of reflux events >80 or positive symptom association indices (SI or SAP). AET values were calculated from the 24-hour monitoring period and therefore represent overall acid exposure during the complete recording duration.

Proximal reflux episodes (defined as reflux events reaching the proximal esophageal impedance channels) were recorded as a measure of reflux extent; however, no standardized pathological threshold was applied, and pathological reflux was defined primarily according to distal AET criteria.

Independent variables included demographic and clinical characteristics such as sex, age, body mass index (BMI), comorbidities, presence of hiatal hernia on endoscopy, typical esophageal symptoms, and atypical (extra-esophageal) symptoms.

Data were retrospectively extracted from the institutional registry database and cleaned according to the predefined inclusion and exclusion criteria. The dataset was imported into R software (version 4.2.2) using the RStudio interface (version 2022.07.02). Four outliers in age and weight were identified and replaced using median imputation.

### Statistical analysis

Descriptive statistics were used to summarize baseline characteristics. Continuous variables were assessed for normality using the Shapiro–Wilk test. As most variables were not normally distributed, non-parametric methods were applied.

An initial univariable association analysis was conducted by estimating crude odds ratios (ORs) between the outcome variable and each candidate predictor, prior to the specification of the multivariable logistic regression model. For categorical variables, associations were additionally assessed using the Chi-square test, while continuous variables were compared using the Wilcoxon rank-sum test. Homogeneity of variances was evaluated using the Fligner–Killeen test.

Variables considered clinically relevant and statistically significant in univariable analyses were included in the multivariable logistic regression model.

BMI was primarily modeled as a continuous variable to preserve statistical information. Sensitivity analyses were performed using categorical BMI thresholds (≥25 and ≥30 kg/m^2^) to evaluate the robustness of the model.

Variables with non-significant confidence intervals were evaluated in sensitivity analyses and excluded from the final multivariable model to improve interpretability.

Potential collinearity between age and hiatal hernia was evaluated using variance inflation factors (VIF). Sensitivity analyses were also performed excluding age from the model to assess the stability of the estimates.

Multicollinearity for all variables was assessed using VIF. Model discrimination was evaluated using receiver operating characteristic (ROC) curves, and calibration was assessed using the Hosmer–Lemeshow goodness-of-fit test. The Breusch–Pagan test was additionally used to assess model specification.

## RESULTS

A total of 2688 patients with suspected LPR were included in the study. Only 226 of them (8.4%) were found to have true pathological gastroesophageal reflux. Table 1 show the sociodemographic and clinical characteristics considered in the analyzed sample, stratified according to normal versus pathological reflux.

**Table 1:** Clinical and sociodemographic characteristics of patients with suspected laryngopharyngeal reflux according to the diagnosis of pathological reflux. *N* = 2688 patients

**Variables**	**GER**		***P*-value**
	**Normal**	**Abnormal**	
**Sociodemographic characteristics**			
Gender			
F Gender, *n* (%)	1829 (74.2)	149 (65.9)	**0**.**006**
M Gender, *n* (%)	633 (25.7)	77 (34.1)	**0**.**006**
Body mass index mean (SD)	25.2 (3.89)	26.2 (3.29)	**0**.**000**
Age mean (SD)	52.8 (13.8)	56.2 (13.9)	**0**.**000**
**Symptoms, *n* (%)**			
Heartburn	1655 (67.2)	177 (78.3)	**0**.**001**
Cough	1350 (54.8)	128 (56.6)	0.602
Regurgitation	1577 (64.0)	166 (73.4)	**0**.**005**
Chest pain	1089 (44.2)	109 (48.2)	0.247
Dysphagia	862 (35.0)	86 (38.0)	0.360
Coughing	1768 (71.8)	167 (73.8)	0.505
Epigastralgia	1496 (60.7)	143 (63.2)	0.459
Bitter taste	1335 (54.2)	119 (52.6)	0.650
Globus	1353 (54.9)	123 (54.4)	0.878
Dysphonia	431 (17.5)	45 (19.9)	0.365
Belching	1034 (41.9)	90 (39.8)	0.526
Hiccups	184 (7.47)	21 (9.29)	0.324
**Comorbidities, *n* (%)**			
Diabetes	79 (3.20)	4 (1.76)	0.231
High blood pressure	383 (15.5)	39 (17.2)	0.501
Asthma	80 (3.24)	9 (3.98)	0.556
Hypothyroidism	312 (12.6)	33 (14.6)	0.407
**Test results**			
Hiatal hernia, n (%)	492 (19.9)	88 (38.9)	**0**.**000**
Acid exposure time, mean (SD)	0.8 (1.3)	13.2 (14.4)	0.000
Distal eeflux episodes, mean (SD)	46 (29.1)	93 (52)	0.000
Proximal reflux episodes, mean (SD)	25.9 (21.2)	50 (29.8)	0.000
**Total patients**	2462	226	

The univariable analysis showed a significant association with pathological reflux for higher BMI, male sex, older age, heartburn, regurgitation, and hiatal hernia (Table 1). Except for regurgitation, these same variables also demonstrated significant associations in the multivariate model used in the study (Table 2). The variables that demonstrated association with abnormal reflux in the multivariate analysis were hiatal hernia (OR 2.30), male sex (OR 1.68), heartburn (OR 1.68), age >59 years (OR 1.46), increased BMI (OR 1.06).

**Table 2:** Factors associated with pathological gastroesophageal reflux in a cohort of 2688 patients: multivariable analysis

**Predictors**	**Odds** **ratios**	**Std.** **error**	**CI**	**Statistic**	***P*-value**
(Intercept)	0.01	0.00	0.00–0.02	−9.82	<0.001
Age > 59.4 yrs	1.46	0.22	1.08–1.98	2.45	0.014
Increased BMI	1.06	0.02	1.03–1.10	3.41	0.001
Heartburn	1.68	0.29	1.21–2.37	3.03	0.002
Hiatal hernia	2.30	0.34	1.72–3.07	5.63	<0.001
Male sex	1.68	0.26	1.25–2.26	3.43	0.001

As shown in Table 1, a comparison of symptom profiles between groups showed that patients with pathological reflux were more likely to report typical reflux symptoms, including heartburn (78.3% vs 67.2%, *P* = 0.001) and regurgitation (73.4% vs 64.0%, *P* = 0.005). In contrast, atypical or laryngopharyngeal symptoms—including cough, dysphagia, globus sensation, dysphonia, and others—did not differ significantly between patients with and without pathological GERD (all *P* > 0.05).

Sensitivity analyses using categorical BMI thresholds (≥25 and ≥30 kg/m^2^) showed similar model discrimination, supporting the stability of the BMI effect. Sensitivity analyses excluding age from the model showed similar model discrimination, supporting the stability of the multivariable model.

**Fig. 1:**
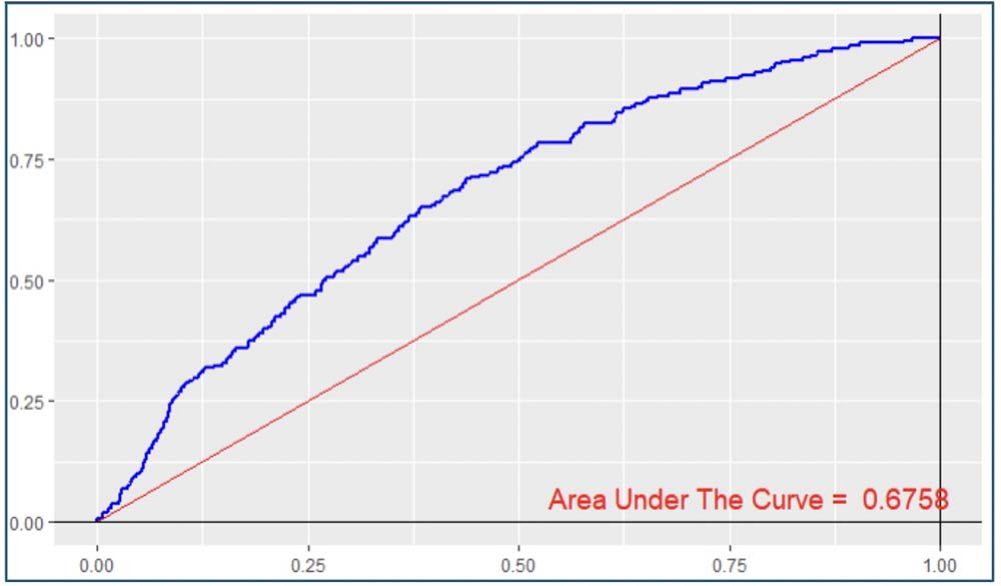
Diagnostic performance of clinical predictors for pathological gastroesophageal reflux: ROC analysis in 2688 patients with laryngopharyngeal reflux symptoms.

According to the ROC curve, the area under the curve was 0.676, corresponding to a predictive value of 67.6% (Fig. 1). In other words, among patients with clinical suspicion of LPR who exhibit the five previously described variables, the probability of confirming a diagnosis of abnormal reflux is 67.6%.

**Fig. 2:**
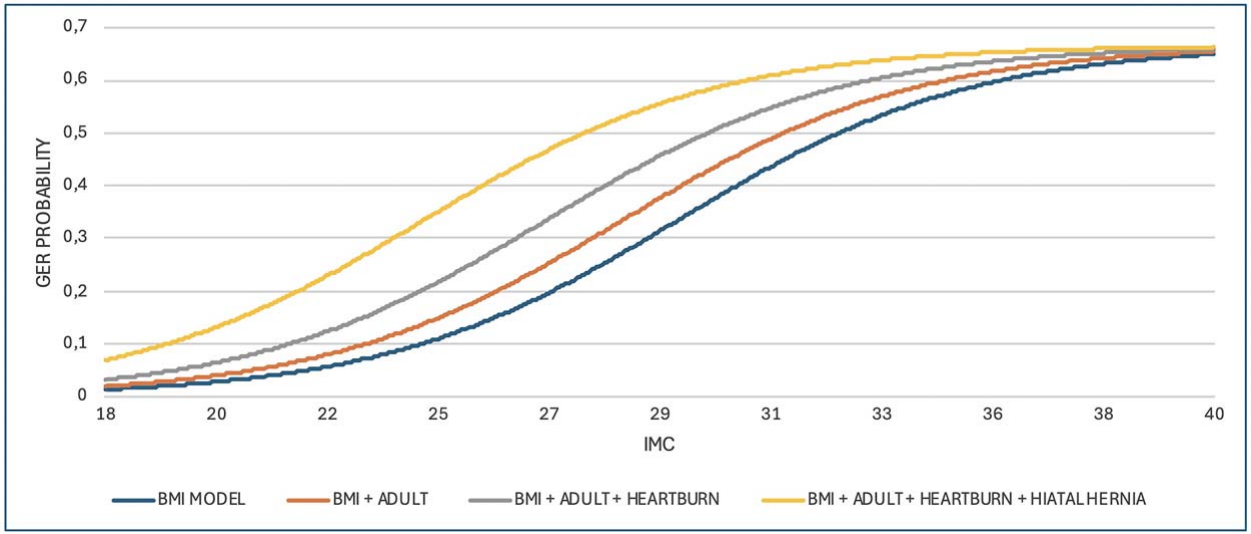
Incremental predictive value of clinical risk factors for pathological gastroesophageal reflux: additive risk modeling based on multivariable analysis. *N* = 2688 patients.

Finally, an analysis was performed to calculate the probability of abnormal reflux based on the cumulative addition of the model’s predictive variables. This was done to determine the effect on risk when individuals present more than one predictive factor. The results of this analysis are observed in Fig. 2. There is a linear increase in the risk of abnormal reflux associated with increasing BMI, and a marked increase in risk when age >59 years, heartburn, and hiatal hernia are added sequentially.

## DISCUSSION

The association between clinical variables and a positive pH-impedance result for pathological gastroesophageal reflux in patients referred for suspected LPR was evaluated in a large cohort of 2688 individuals. Notably, only 8.4% of patients demonstrated objective evidence of pathological reflux, highlighting a marked discordance between laryngopharyngeal symptoms and confirmed GERD. This finding reinforces growing concerns regarding the potential overdiagnosis of LPR when based solely on nonspecific symptomatology.

LPR, as part of the extraesophageal spectrum of GERD, has gained increasing attention as a potential cause of upper airway symptoms.^3^^,^^8^ However, its diagnosis remains challenging due to the absence of a true gold standard and the nonspecific nature of clinical manifestations.^6^ As a result, many patients are empirically diagnosed and treated despite lacking objective evidence of reflux, contributing to unnecessary healthcare utilization. This diagnostic uncertainty is compounded by an incomplete understanding of its pathophysiology. While LPR shares proposed mechanisms with GERD—such as lower esophageal sphincter dysfunction, esophageal acid exposure, and vagally mediated responses—these mechanisms have not been conclusively established in all patients.^1^^,^^6^

A key finding of this study is the marked discordance between laryngopharyngeal symptoms and objectively confirmed GERD. As previously mentioned, although all patients were referred for suspected LPR, only a small proportion demonstrated pathological reflux on pH-impedance monitoring. Moreover, patients with pathological GERD exhibited a clinical phenotype consistent with that typically observed in gastroenterology practice. In contrast, prior studies have shown that patients with isolated laryngopharyngeal symptoms may not share traditional GERD risk factors, such as obesity.^9^ These findings highlight the limited diagnostic value of throat symptoms alone and support a more cautious interpretation of LPR, with consideration of alternative etiologies.^10^ In line with the San Diego Consensus, when evaluating a patient with suspected LPR, a comprehensive assessment of esophageal function should include objective testing to confirm or exclude pathological reflux, such as upper endoscopy and esophageal pH monitoring.^11^

From a pathophysiological perspective, these findings support a multifactorial origin of laryngopharyngeal symptoms, in which reflux may represent only one of several contributing factors.^3^ Alternative mechanisms—including functional disorders, airway hypersensitivity, inflammatory conditions, and non-reflux-related etiologies—likely play a significant role in symptom generation.^5^ Even in patients with documented reflux, the causal relationship between reflux events and laryngeal symptoms remains uncertain, particularly in the absence of abnormal distal acid exposure^2^^,^^12^

The most important predictor identified was hiatal hernia (OR 2.30, 95% CI [1.72–3.07]), likely reflecting disruption of the physiological antireflux barrier due to migration of the lower esophageal sphincter.^1^ Additional factors associated with pathological reflux included male sex (OR 1.68, 95% CI [1.25–2.26]), presence of heartburn (OR 1.68, 95% CI [1.21–2.37]), age greater than 59 years (OR 1.46, 95% CI [1.08–1.98]), and increased BMI (OR 1.06, 95% CI [1.03–1.10]). These findings are consistent with established GERD epidemiology and support the role of traditional reflux-related factors in identifying patients with objective disease.

A logistic regression model incorporating these variables demonstrated a predictive value of 67.6%. Age was considered a confounding variable and required stratification. Dichotomization using a decision-tree approach yielded a cutoff of 59.4 years, above which the probability of pathological reflux increased significantly. These findings support a risk-based approach to identifying patients more likely to benefit from objective reflux testing.

In contrast, laryngopharyngeal symptoms—including cough, dysphonia, globus sensation, throat clearing, hiccups, epigastric pain, and belching—were not associated with pathological reflux. These results reinforce the limited sensitivity and specificity of extraesophageal symptoms for GERD and further support a more cautious interpretation of symptom-based diagnoses.

Our findings are consistent with the recently proposed COuGH RefluX score by Krause *et al.*, an international multicenter model developed and externally validated in patients with chronic laryngeal symptoms. This score incorporates clinical variables such as cough, overweight/obesity, globus sensation, hiatal hernia, regurgitation, and male sex, and demonstrated an equal discriminative performance (AUC 0.67). Notably, its performance was lower in patients with isolated laryngeal symptoms and higher in those with concomitant esophageal symptoms.^13^

In line with these findings, our model also identified traditional GERD-related factors—such as male sex, increased BMI, and hiatal hernia—as the main predictors of pathological reflux, while isolated laryngopharyngeal symptoms showed limited diagnostic value. Although differences exist in outcome definition and study design, both models support the concept that objective GERD in patients with suspected LPR is better predicted by typical GERD features than by isolated extraesophageal manifestations.

The clinical relevance of isolated proximal reflux episodes in patients with normal distal acid exposure remains uncertain and should be interpreted cautiously. These findings further indicate that atypical or laryngopharyngeal symptoms do not reliably discriminate patients with objectively confirmed GERD, in contrast to typical reflux symptoms.

Some limitations should be considered when interpreting these findings. pH-impedance monitoring is a catheter-based technique that may not fully capture day-to-day variability in reflux patterns, potentially resulting in false-negative findings. Additionally, information on prior PPI therapy and patients who did not complete reflux testing was not systematically captured.

From a methodological standpoint, its conduct at a single referral center may introduce selection bias, as the population likely represents patients with a high pretest probability of LPR. Symptom characterization was not uniformly performed using validated instruments, potentially introducing measurement variability. Additionally, the predictive model lacks external validation. Despite these limitations, this study has notable strengths, including a large sample size, the use of objective reflux monitoring, and the evaluation of clinically relevant predictors in a real-world population.

## CONCLUSIONS

Only a small proportion of patients referred for suspected LPR demonstrated objective evidence of GERD, with a prevalence of just 8.4% in this cohort. This marked discrepancy underscores the potential overdiagnosis of LPR when based primarily on nonspecific laryngopharyngeal symptoms.

Our findings show that pathological reflux is more accurately predicted by traditional GERD-related factors—such as male sex, increased BMI, hiatal hernia, and typical esophageal symptoms—rather than by isolated extraesophageal manifestations. These results support a more selective and evidence-based approach to diagnosis and management.

In clinical practice, reliance on symptom-based diagnosis alone may lead to unnecessary treatment and overuse of PPI. Therefore, objective reflux testing and careful patient selection should be prioritized, particularly in those with established GERD risk factors. In patients presenting with nonspecific laryngopharyngeal symptoms, broadening the differential diagnosis is essential before attributing symptoms to GERD.

## Notes

Specific author contributions: Luis Fernando Pineda Ovalle (Conceptualization, Data curation, Formal analysis, Funding acquisition, Investigation, Methodology, Project administration, Resources, Software, Supervision, Validation, Visualization, Writing—original draft, Writing—review & editing), Valentina Salcedo (Conceptualization, Data curation, Formal analysis, Investigation, Methodology, Project administration, Resources, Software, Supervision, Validation, Writing—original draft, Writing—review & editing), Alejandra del Pilar Rodríguez (Conceptualization, Data curation, Formal analysis, Investigation, Methodology, Project administration, Resources, Software, Supervision, Validation, Visualization, Writing—original draft, Writing—review & editing), Cristian Andres Mora León (Conceptualization, Data curation, Formal analysis, Investigation, Methodology, Project administration, Resources, Software, Supervision, Validation, Visualization, Writing—original draft, Writing—review & editing), Andrea Gómez Rodríguez (Conceptualization, Data curation, Formal analysis), Laura Moya Valenzuela (Conceptualization, Data curation, Formal analysis), David Páramo Hernández (Conceptualization, Data curation, Formal analysis).

Funding: None declared.
